# Facile Preparation of a Platinum Silicide Nanoparticle-Modified Tip Apex for Scanning Kelvin Probe Microscopy

**DOI:** 10.1186/s11671-015-1096-8

**Published:** 2015-10-15

**Authors:** Chun-Ting Lin, Yu-Wei Chen, James Su, Chien-Ting Wu, Chien-Nan Hsiao, Ming-Hua Shiao, Mao-Nan Chang

**Affiliations:** Instrument Technology Research Center, National Applied Research Laboratories, Hsinchu, 300 Taiwan; Department of Physics, National Chung Hsing University, Taichung, 402 Taiwan; National Nano Device Laboratories, National Applied Research Laboratories, Hsinchu, 300 Taiwan; Institute of Nanoscience, National Chung Hsing University, Taichung, 402 Taiwan

## Abstract

In this study, we propose an ultra-facile approach to prepare a platinum silicide nanoparticle-modified tip apex (PSM tip) used for scanning Kelvin probe microscopy (SKPM). We combined a localized fluoride-assisted galvanic replacement reaction (LFAGRR) and atmospheric microwave annealing (AMA) to deposit a single platinum silicide nanoparticle with a diameter of 32 nm on the apex of a bare silicon tip of atomic force microscopy (AFM). The total process was completed in an ambient environment in less than 3 min. The improved potential resolution in the SKPM measurement was verified. Moreover, the resolution of the topography is comparable to that of a bare silicon tip. In addition, the negative charges found on the PSM tips suggest the possibility of exploring the use of current PSM tips to sense electric fields more precisely. The ultra-fast and cost-effective preparation of the PSM tips provides a new direction for the preparation of functional tips for scanning probe microscopy.

## Background

With the prosperous development of nanotechnology, the demand on nondestructive analysis of the distribution of charge density [1], magnetic field [2], surface potential [3], etc. is surging in nanometrology. In the field of nondestructive analysis, scanning Kelvin probe microscopy (SKPM) has been widely used to analyze the surface potential distribution of materials [4–6]. Spatial resolutions of the geometry and surface potential are both crucial issues in SKPM. Traditionally, apexes of silicon (Si) tips coated with metallic thin film, e.g., platinum-iridium alloy (PtIr), are widely used for SKPM. However, the spatial resolution was seriously limited because of the stray-field generated by the metallic coating [7–9]. To reduce this stray-field phenomenon, many nanostructures have been proposed for tip modifications, including carbon nanotube [10–12], Pt nanowire [13], single metallic nanoparticle (NP) [14, 15], etc. Moreover, for many decades, electron beam induced deposition (EBID) has been a major approach for tip modification [16–19]. However, the vacuum system required for the EBID process and the need to manipulate electron beams make the tip modification costly and inefficient. For these reasons, the development of nonvacuum technology for tip modifications has been presented. Electrodeposition [15] and electroless deposition [14] are both feasible approaches for tip modification under ambient conditions. Recently, we successfully prepared an apex of an Ag NP-modified silicon tip (Ag tip) by utilizing a localized fluoride-assisted galvanic replacement reaction (LFAGRR), which provides a facile and cost-effective process for tip modification [14]. However, the instability of Ag under ambient conditions directly limits the commercialization of the Ag tips [20]. In this paper, the LFAGRR was extended to prepare Pt NP-modified silicon tip apexes (Pt tips). Moreover, we introduced atmospheric microwave annealing (AMA) to further stabilize the tip apexes by transforming the Pt tips into platinum silicide NP-modified tip apexes (PSM tips). Scanning electron microscopy (SEM), transmission electron microscopy (TEM), SKPM, and electrostatic force microscopy (EFM) were employed to study the PSM tips and the corresponding Si-based Pt silicide NPs. Our results demonstrate that the PSM tips benefit from both high spatial resolutions of topography and surface potential. Moreover, the facile and cost-effective approach combining the LFAGRR and AMA provides a new direction for preparing functional tips for scanning probe microscopy.

## Methods

### Preparation of Pt and PSM Tips

**Fig. 1:**
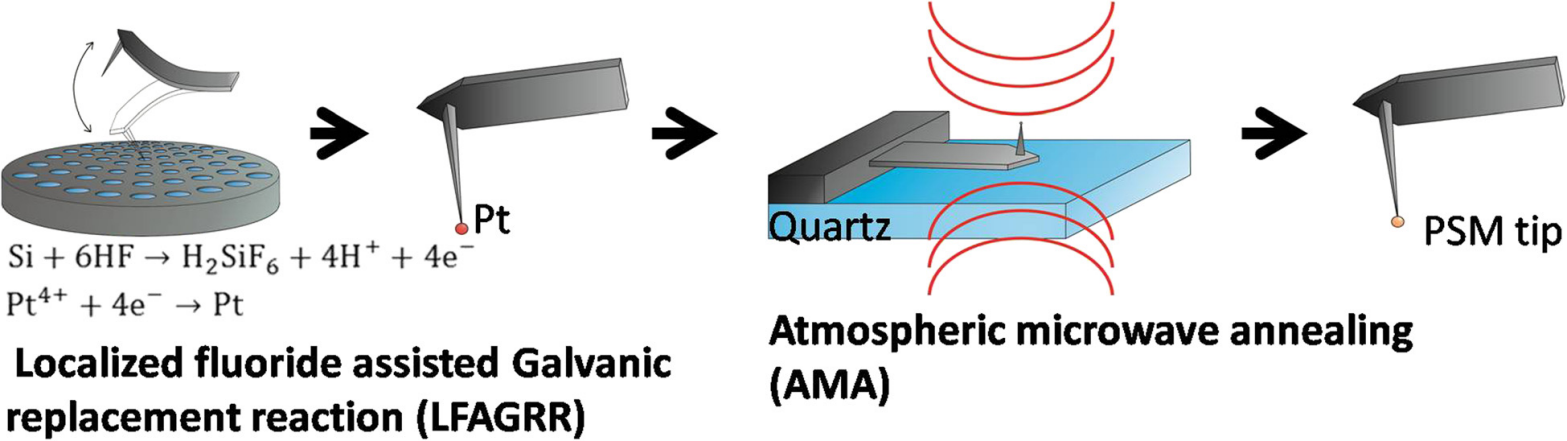
The schema of the fabrication process of a PSM tip

Figure 1 shows the schema of the fabrication process of the PSM tips. In a typical LFAGRR process, we utilized a slice of commercial anodic aluminum oxide (AAO) with 100 nm of pore size (Whatman) as the template for carrying the electrolyte comprising 0.01 M chloroplatinic acid (H_2_PtCl_6_) and 16 % (*v*/*v*) buffered oxide etchant (BOE) (comprising 34 % NH_4_F and 7 % HF). The prepared AAO template was followed by a continuous tapping process for 30 s in the atomic force microscopy (AFM) (Bruker D3100) by utilizing silicon tips (NANOSENSORS) to form Pt NP on the tip apexes, denoted as Pt tips. The prepared tips then underwent the AMA process in a commercial microwave oven (Freser MW-1806) at 1800 W for 60 and 90 s, respectively. The prepared tip apexes were denoted as PSM-60 and PSM-90 tip. We also employed an n-type Si wafer that was dipped in the same electrolyte for various durations and had undergone the same AMA for SEM and TEM analysis.

### Surface Morphology and Material Characterization

SEM (Hitachi S-4000) and TEM (JEOL JEM2010F) were employed to investigate the surface profile of the prepared samples. Energy-dispersive X-ray spectroscopy (EDS) was used to analyze the elemental composition. The SKPM and EFM images were acquired by a scanning probe microscope (Bruker D3100). The SKPM measurement parameters were image size = 2 μm, scanning rate = 0.2 Hz, lift height = 30 nm, V_ac_ = (1.1–1.5) V, and ω_2_ = (31–35) kHz. The EFM measurement parameters were: image size = 1 μm, scanning rate = 0.5 Hz, lift height = 30 nm, and substrate bias = (0, −0.5, −1.0, −1.5) V, respectively. All measurements were performed in constant environment conditions with temperature = (20 ± 0.3) °C and relative humidity = (40 ± 5) % RH. All of the chemicals used in the experiment were reagent grade.

## Results and Discussion

**Fig. 2:**
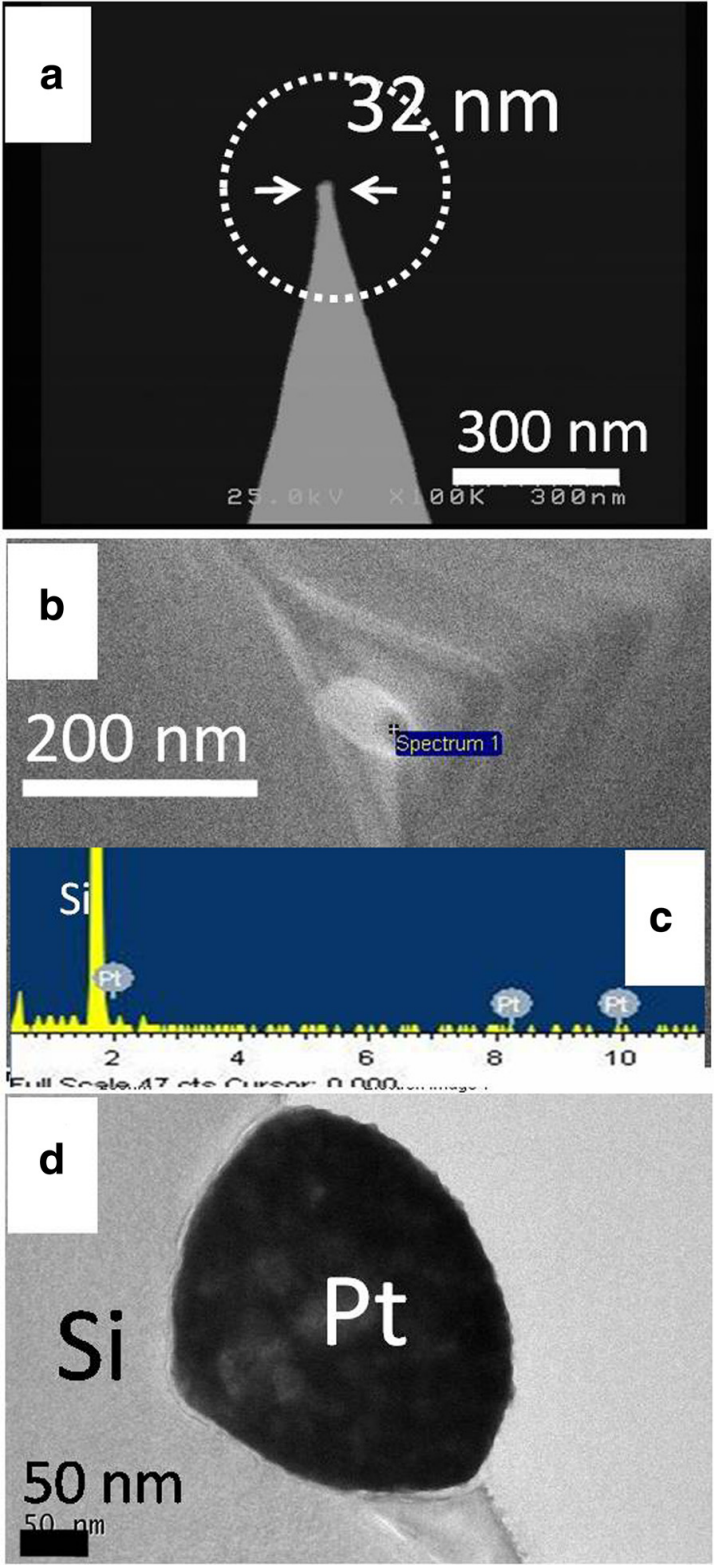
SEM, EDS, and TEM results. **a**, **b** The SEM images of Pt NP-modified silicon tip apex. **c** The corresponding EDS analysis of **b**. **d** The TEM image of Pt NPs grown on planar Si using fluoride-assisted galvanic replacement reaction. The electrolyte for Pt deposition is composed of 0.01 M of H_2_PtCl_6_ and 16 % (*v*/*v*) buffered oxide etchant (BOE). The duration of deposition for the silicon tip is 30 s; for the planar silicon, it is 300 s

**Fig. 3:**
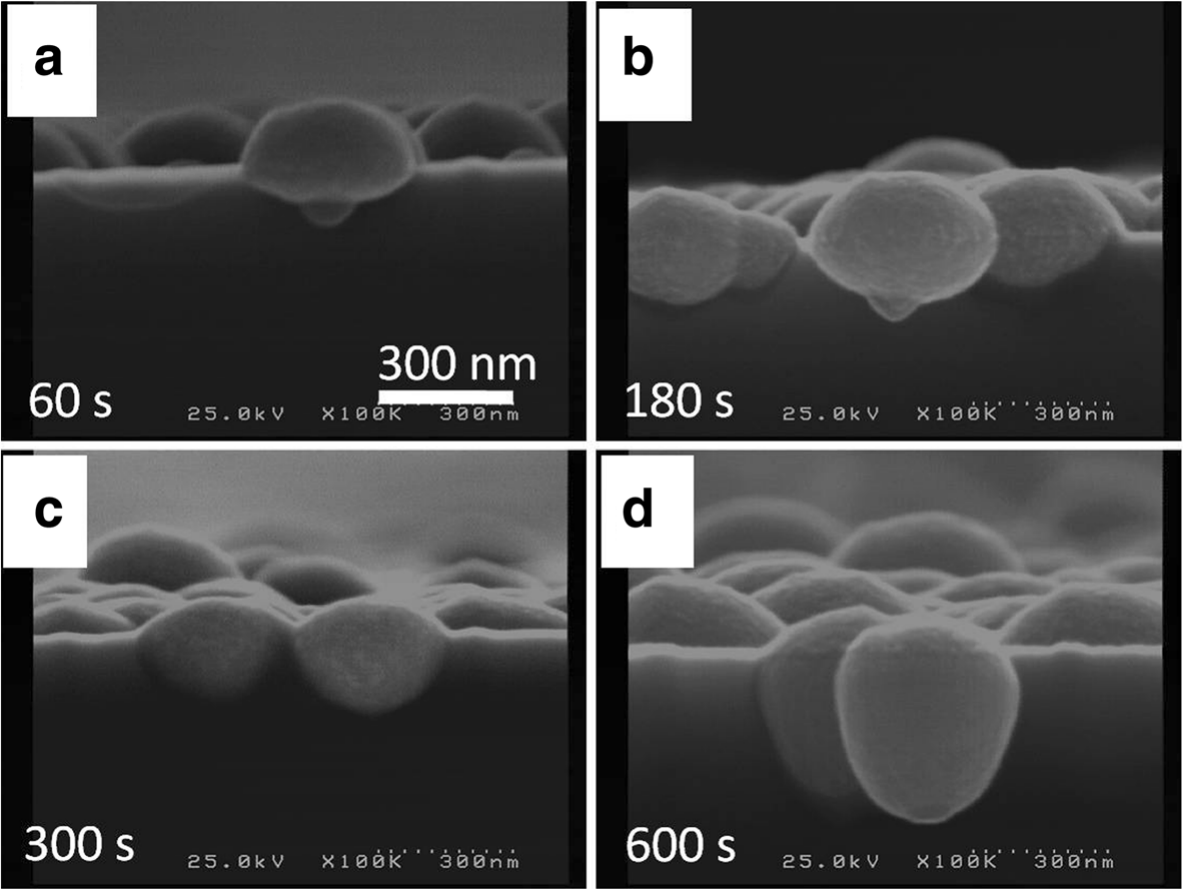
The SEM images of Pt NP-modified planar silicon prepared by FAGRR for various reaction durations. The electrolyte for Pt deposition is composed of 0.01 M of H_2_PtCl_6_ and 16 % (*v*/*v*) buffered oxide etchant (BOE)

Figure 2a, b shows the typical Pt tip prepared by the LFAGRR before conducting the AMA process. A Pt NP with a 32 nm diameter can be clearly observed, which coincides with our previous report about Ag tips [14]. The ingredient of the Pt tip was verified using EDS (Fig. 2c). Figure 2d shows the TEM image of Pt NP-modified Si base prepared by the fluoride-assisted galvanic replacement reaction (FAGRR). The pinecone-like Pt NPs were found embedded into the Si domain, which agrees with the previous Pt nanostructures reported by Ye et al. [21]. We also noted that the Pt NPs elongated and embedded more into the Si domain as the duration of the reaction increased (Fig. 3).

**Fig. 4:**
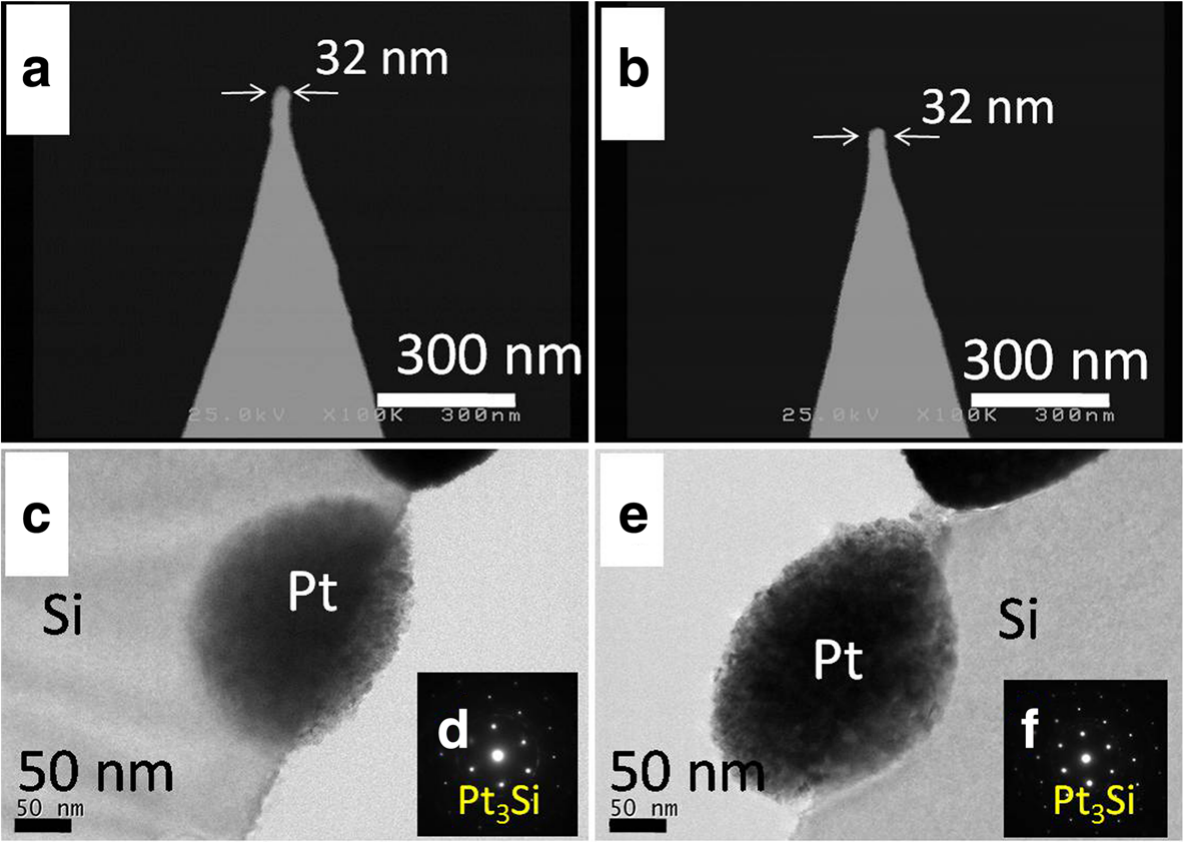
SEM, TEM, and SAD results. **a**, **b** The SEM images of PSM tips. **c**, **e** The TEM images of Pt NP-modified planar silicon after AMA. **d**, **f** The SAD pattern from **c** and **e**, respectively. The AMA duration was 60 s for **a**, **c**, and **d** and 90 s for **b**, **e** and **f**, respectively. The power of the microwave was 1800 W

Figure 4 shows the SEM images of typical PSM tips (Fig. 4a, b) and the TEM images of the corresponding Pt NP-modified Si wafer obtained after it was exposed to various AMA conditions (Fig. 4c, e). The morphology of the tip apexes was unchanged compared to that before exposure to AMA conditions (Fig. 2a). However, an obvious interface diffusion was observed between Pt and Si in the TEM images (Fig. 4c, e). Moreover, the formation of Pt_3_Si was verified by the selected area diffraction (SAD) in the TEM (Fig. 4d, f). Note that microwave annealing is a common process in the semiconductor field [22, 23]. However, few previous studies report the formation of Pt silicide using microwave annealing. Thermal annealing is commonly used in the preparation of Pt silicide-modified AFM tips [24, 25]. Besides, Chou et al. reported the formation of platinum silicide by annealing the junction between silicon carbide and Pt [26]. Takahashi et al. utilized silane to react with Pt at 250 to 400 °C to form platinum silicide [27]. Wang et al. prepared Pt silicide by thermal annealing of a Pt/SiO_2_ junction in a hydrogen environment [28]. A similar form of hydrogen annealing has also been adopted by Liu et al. to prepare Pt_3_Si for benzene oxidation [29]. Compared to these efforts on thermal annealing, our AMA process provides a facile and cost-effective process for the preparation of Pt silicide.

**Fig. 5:**
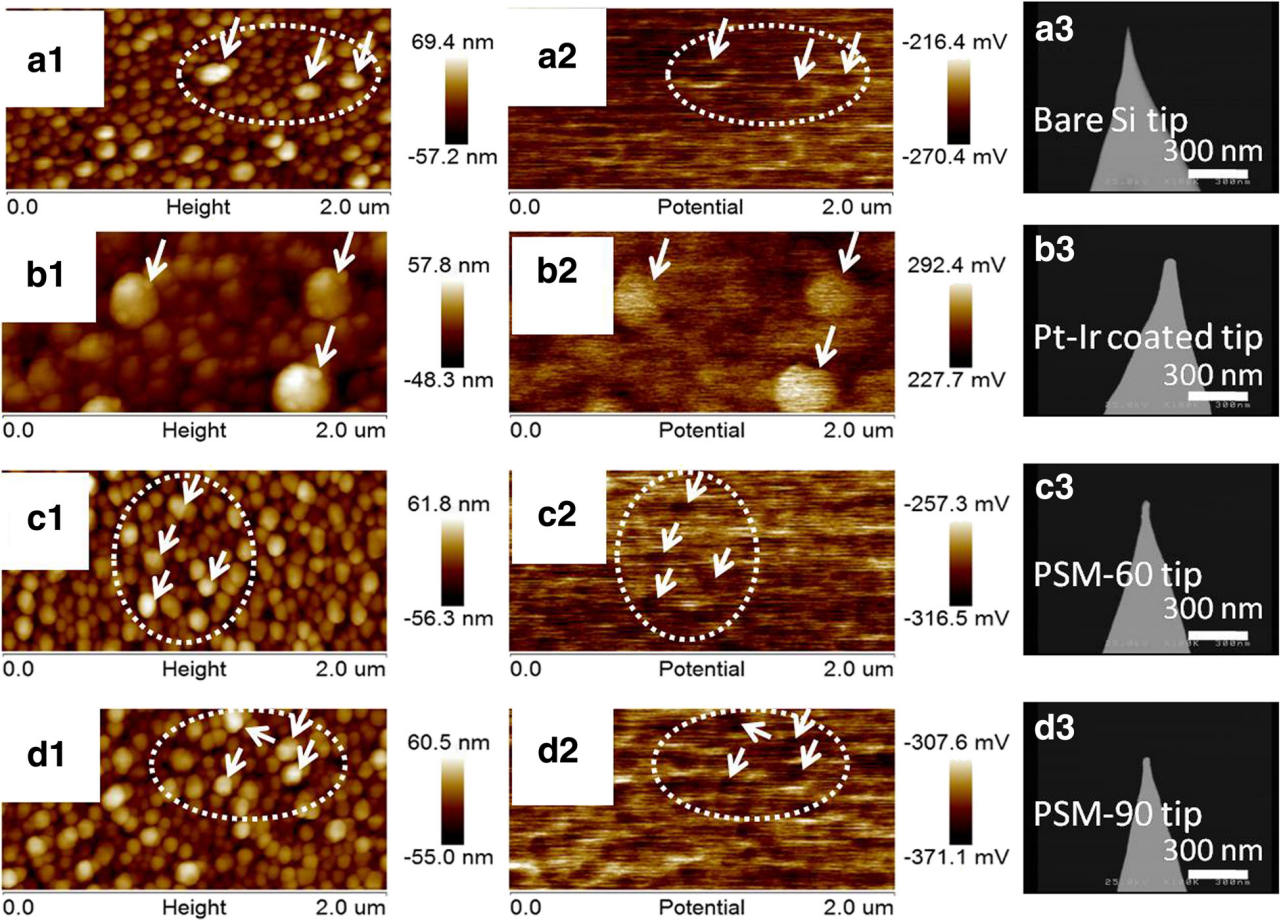
The SKPM images of Si-based Ag nanoislands and the SEM images of the corresponding tips used in the measurement. The SKPM images of Si-based Ag nanoislands acquired by using a bare Si tip (**a1**, **a2**), PtIr-coated Si tip (**b1**, **b2**), PSM-60 tip (**c1**, **c2**), and PSM-90 tip (**d1**, **d2**). The SEM images for the bare Si tip (**a3**), PtIr-coated Si tip (**b3**), PSM-60 tip (**c3**), and PSM-90 tip (**d3**)

Figure 5 shows the SKPM images of Si-based Ag NPs acquired using a bare silicon tip (Fig. 5a1, a2), a commercial PtIr-coated silicon tip (Fig. 5b1, b2), a PSM-60 tip (Fig. 5c1, c2), and a PSM-90 tip (Fig. 5d1, d2), respectively. Figure 5a3, b3, c3, d3 are the SEM images of the corresponding tips used in the SKPM measurements. The SKPM images obtained using PSM tips demonstrate clear potential contrast compared to that obtained by either commercial PtIr-coated Si or bare Si tips. Moreover, the resolution of topography of AFM images acquired using PSM tips stayed nearly the same as that of images acquired using a bare Si tip. The results in Fig. 5 revealed that PSM tips are capable of enhancing the spatial resolution of surface potentials while maintaining the resolution of topography. It is noted that the commercial PtIr-coated Si tips require vacuum based processes, e.g., sputter or e-beam assisted deposition. So that the PtIr-coated Si tips suffer from high cost and the sacrifice of sharpness at the tip apexes. In contrast, our PSM tips can be fabricated under ambient conditions in less than 3 min by combining LFAGRR and AMA. The localized electroless deposition maintains the sharpness of the tip apexes. Also, the vacuum-free fabrication prominently reduces the cost of the PSM tips. In addition, the negative shift of the surface potential found on the SKPM profiles collected using PSM tips suggests that the work function of the tip apexes varied with the AMA process, which could be attributed to the formation of Pt_3_Si that varies the work function of the tip apex.

**Fig. 6:**
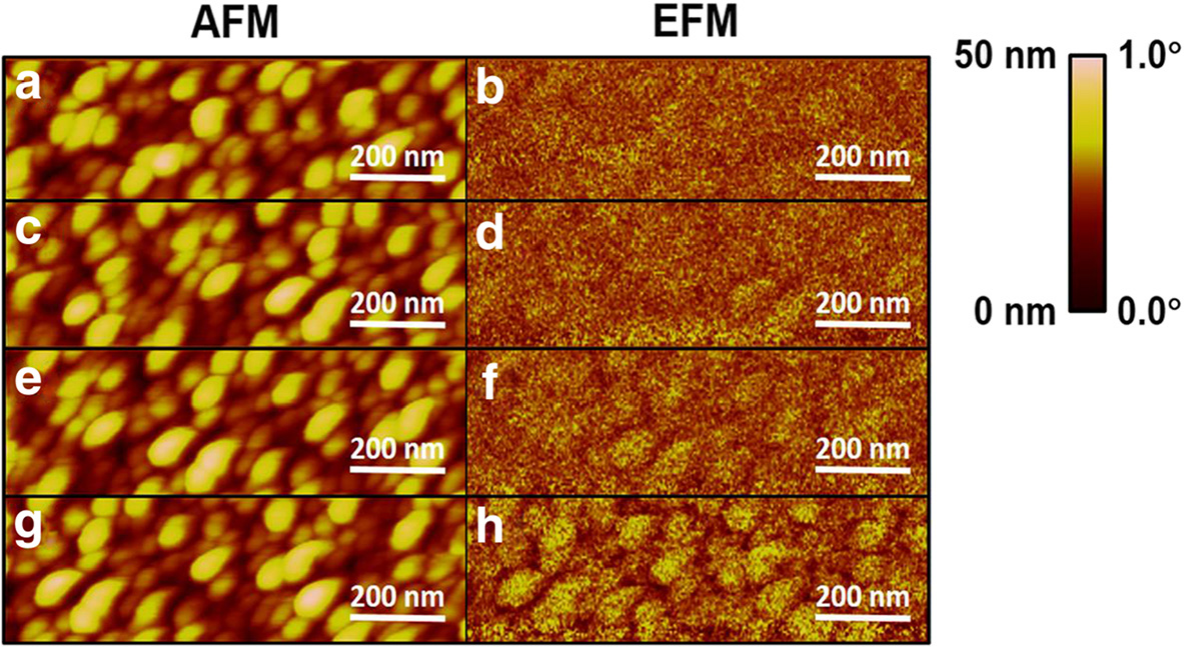
The EFM images of Si-based Au nanoislands acquired by using a PSM-60 tip for various applied biases on the substrate. The applied bias was 0 V for **b**, −0.5 V for **d**, −1.0 V for **f**, and −1.5 V for **h**. **a**, **c**, **e**, **g** The corresponding topographies acquired by applying AFM to **b**, **d**, **f**, and **h**, respectively

Besides the enhanced spatial resolution of the surface potential, the PSM tip was negatively charged. Figure 6 shows the EFM images of Si-based Au nanoislands under various biases. No phase difference was found when zero bias was applied to the Au nanoislands. When the substrate of the Au nanoislands was subjected to a more negative bias, the phase difference became clearer, which means that the repulsive force between the PSM tip and the Au islands became stronger. That phenomenon provided direct evidence that the PSM tip was negatively charged. The result could be attributed to the formation of Pt silicide that reduces the Schottky barrier and enables the electron injection from the domain of n-type Si to platinum [24, 30, 31]. Our results demonstrated the possibility of engineering tip apexes with confined charges for more precise sensing of electrical fields. In addition, the ultra-fast and cost-effective preparation of PSM tips provides a new direction for preparing functional tips for scanning probe microscopy.

## Conclusions

In summary, we proposed an ultra-facile process combining LFAGRR and AMA to prepare PSM tips under ambient conditions. The improved potential resolution of Si-based Ag nanoislands in SKPM measurement was realized. Moreover, the resolution of the topography was comparable to that of a bare silicon tip. In addition, the negative charges found on the PSM tips suggests the possibility of exploring the use of current PSM tips to sense electric fields more precisely. The ultra-fast and cost-effective preparation of the PSM tips provides a new direction for preparing functional tips for scanning probe microscopy.
